# Platinum Clusters on Vacancy-Type Defects of Nanometer-Sized Graphene Patches

**DOI:** 10.3390/molecules17077941

**Published:** 2012-07-02

**Authors:** Takashi Yumura, Tatsuya Awano, Hisayoshi Kobayashi, Tokio Yamabe

**Affiliations:** 1 Department of Chemistry and Materials Technology, Kyoto Institute of Technology, Matsugasaki, Sakyo-ku, Kyoto 606-8585, Japan; 2 Nagasaki Institute of Applied Science, 536 Aba-machi, Nagasaki 851-0193, Japan

**Keywords:** density functional theory, graphene, cluster, catalyst, spin state

## Abstract

Density functional theory calculations found that spin density distributions of platinum clusters adsorbed on nanometer-size defective graphene patches with zigzag edges deviate strongly from those in the corresponding bare clusters, due to strong Pt-C interactions. In contrast, platinum clusters on the pristine patch have spin density distributions similar to the bare cases. The different spin density distributions come from whether underlying carbon atoms have radical characters or not. In the pristine patch, center carbon atoms do not have spin densities, and they cannot influence radical characters of the absorbed cluster. In contrast, radical characters appear on the defective sites, and thus spin density distributions of the adsorbed clusters are modulated by the Pt-C interactions. Consequently, characters of platinum clusters adsorbed on the sp^2^ surface can be changed by introducing vacancy-type defects.

## 1. Introduction

Graphitic carbon materials serve as a support material [1] for anode catalysts such as platinum clusters in proton exchange membrane (PEM) fuel cells [2,3,4,5,6,7,8,9]. The supported Pt clusters catalyze the activation of hydrogen molecules to form protons and electrons on the anode of fuel cells. Consequently, the size of Pt clusters is a crucial parameter in determining their catalytic activity. Actually, clusters of less than 3 nm are more effective for catalyzing the H_2_ dissociation [9]. During the catalytic reactions, adjacent clusters tend to coalescence, forming larger clusters. Accordingly, their catalytic activity decreases as the reaction proceeds. To retain the catalytic activity of supported Pt catalysts, a plausible approach is to strengthen interactions between Pt clusters and underlying carbon sp^2^ surface.

**Figure 1:**
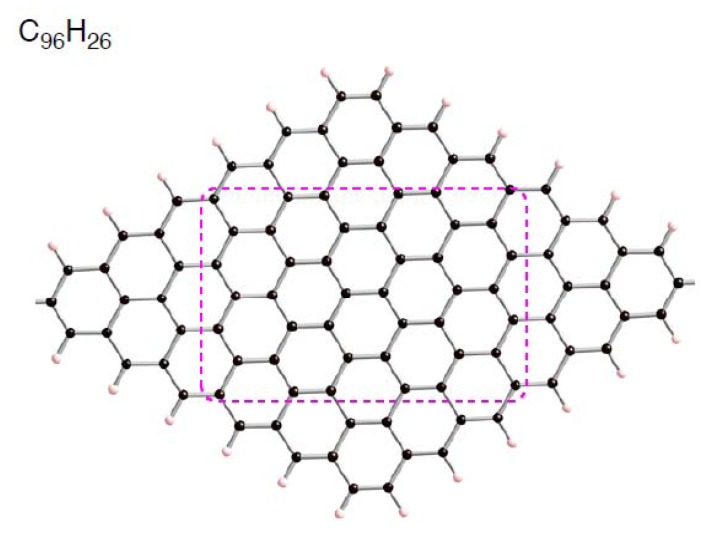
Optimized structure for C_96_H_26_graphene patch.

To come up with a strategy for constructing carbon supports suitable for Pt catalysts, computational simulations are becoming a powerful tool [9,10,11,12,13,14,15,16,17,18,19,20,21,22,23,24,25,26,27,28,29,30,31]. Several computational studies suggest that disrupting the sp^2^ surface by introducing defects (vacancy-type and Stone-Wales type) [9,18,21,25,26,29,30,31], dopants (nitrogen or boron impurities) [14,15,16], and mechanical strain [25] can enhance the interactions with Pt clusters. With respect to the formation of the vacancy-type defects, recent high-resolution transmission electron microscopy (TEM) studies [32,33,34,35] show that electron irradiation of graphene creates vacancy-type defects by removing a few carbon atoms from the surface. After these events, unsaturated carbon atoms are generated. Some of the unsaturated atoms make a covalent bond with an adjacent atom to form a five-membered ring, whereas the others remain two-coordinated. These carbon atoms, which cannot be seen in pristine graphene, are more chemically reactive, and thus they serve as sites for strong adsorption of Pt clusters. Reactive carbon atoms can be also found on edges of zigzag-nanoribbons and zigzag-graphene patches, because their frontier orbital coefficients are located on edge carbon atoms [36,37,38,39,40,41,42]. Thus, one can utilize such reactive edge atoms to trap well Pt clusters [22,27]. Previously, we investigated by means of density functional theory (DFT) calculations how a Pt cluster is bound to the nanometer-size rhombic sp^2^ patch with zigzag-edges (C_96_H_26_) (Figure 1) [22]. Such H-terminated sp^2^ patches are contained in activated carbons as condensed-aromatic-ring fractions [43]. The DFT calculations found that a Pt_6_ cluster preferentially binds into edge atoms of C_96_H_26_ rather than into center atoms. In fact, the Pt_6_ additions to edge atoms were about 50 kcal/mol more stable than those to center atoms [22].

Another interesting feature of nanometer-size graphenes is that they have radical character in the ground state, depending on their shape and size [44,45,46,47,48,49,50,51]. Thus, we assume that interactions between a Pt cluster and a radical sp^2^ patch can modulate the catalytic activity of the supported cluster due to the onset of unpaired electrons on Pt atoms. Based on the assumption, the current study will focus on whether spin states of C_96_H_26_ support have a power to influence the properties of the adsorbed Pt clusters. Furthermore, we are interested in how introduction of vacancy-type defects on the radical C_96_H_26_ support changes the electronic properties of the surface. These changes would have an impact on determining the properties of Pt clusters adsorbed on the sp^2^ support. To increase our understanding of the interactions between a radical sp^2^ support and a Pt cluster, we performed density functional theory (DFT) calculations. The main aim in the current DFT study is to clarify how different electronic properties of C_96_H_26_ support with or without vacancy-type defects influence the interactions with Pt clusters, and concomitantly the properties of the absorbed clusters.

## 2. Results and Discussion

### 2.1. Platinum Clusters on C_96_H_26_ in the Triplet State

**Figure 2:**
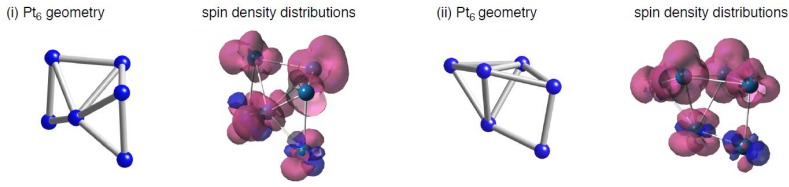
Optimized structures for bare Pt_6_ cluster, and their spin density distributions. Isosurface α- and β-spins are given by pink and blue, respectively.

**Figure 3:**
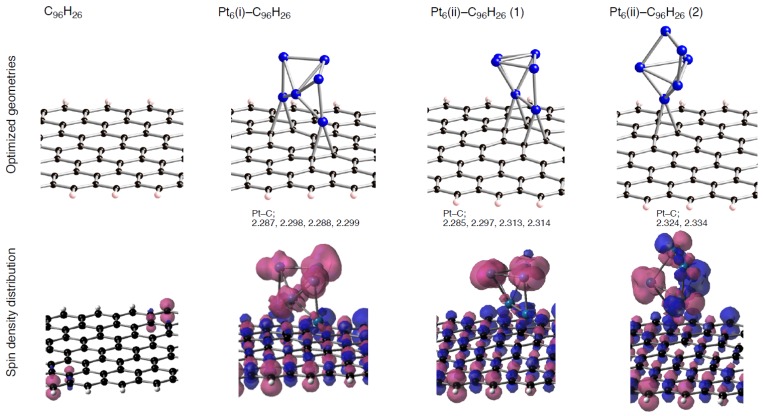
Spin density distributions of optimized C_96_H_26_ and Pt_6_-C_96_H_26_ configurations in the triplet state. Parts of the optimized geometries, corresponding to the region surrounded by pink hashed lines in Figure 1, are given. Isosurface α- and β-spins are given by pink and blue, respectively. Optimized bond lengths are in Å.

To obtain a basic insight on how different spin states of C_96_H_26_ patch affect the interactions with Pt clusters, we investigated how Pt_6_ clusters bind into the sp^2^ surface. Following the previous study [22], two types of Pt_6_ cluster were considered, denoted by (i) and (ii) in Figure 2. The DFT calculations found that their triplet states are energetically stable relative to the corresponding singlet states. The stability of spin-polarized states in Pt clusters was also reported by other groups [52,53,54]. We obtained three optimized geometries for Pt_6_(i) or Pt_6_(ii) clusters adsorbed on C_96_H_26_ (Pt_6_-C_96_H_26_) in the singlet and triplet states. Within the three optimized geometries in Figure 3, one is that the Pt_6_(i) cluster makes four Pt-C bonds with the sp^2^ surface, and the other two are distinguished by whether the number of Pt-C bonds formed between the Pt_6_(ii) cluster and the surface is 2 or 4. In these geometries, optimized lengths of Pt-C bonds range from 2.288 to 2.334 Å.

We estimated in Table 1 the energy difference between the triplet and singlet spin states in each configuration, Δ*E*_state_(Pt_6_-C_96_H_26_), defined as [*E*_total_(triplet state) – * E_total_*(singlet state)] where *E*_total_(triplet state) or *E*_total_(singlet state) is the total energy in each state. As shown in Table 1, the three configurations have negative Δ*E*_state_(Pt_6_-C_96_H_26_) values. These negative Δ*E*_state_(Pt_6_-C_96_H_26_) values indicate that the triplet state of a Pt_6_-C_96_H_26_ configuration is energetically favorable relative to the singlet state, irrespective of the cluster shapes. Furthermore, we see from Table 1 more significant Δ*E*_state_ values in the Pt_6_-C_96_H_26_ configurations than those in the bare Pt_6_ clusters. Thus, the Pt-C interactions influence relative stability of the triplet to singlet states of the Pt_6_ clusters.

Spin density distributions in the triplet Pt_6_-C_96_H_26_ structures are also displayed in Figure 3, where isosurface α- and β-spins are given by pink and blue, respectively. As shown in Figure 3, the Pt-C interactions induce spin densities on C_96_H_26_, although the stable triplet state of pristine C_96_H_26_ has radical characters only on edge carbon atoms. Likewise, we see spin densities on the adsorbed Pt cluster in the configurations. Basically their spin density distributions are similar to those in the bare Pt_6_ clusters (Figure 2), but spin densities slightly decrease on Pt atoms that participates the Pt-C bond formation. The similarity between Pt clusters with and without the carbon support is understandable, because underlying carbon atoms do not have radical characters in pristine C_96_H_26_ in the stable triplet state, and thus they cannot perturb the spin density distributions of Pt clusters even though they interact substantially.

**Table 1:** Energy difference between the singlet and triplet states in Pt_6_-C_96_H_26_ (ΔE_state_ in kcal/mol) ^a^.

	Pt_6_(i)	Pt_6_(ii)-(1)	Pt_6_(ii)-(2)
**Bare clusters**	–14.5	–5.4	–5.4
**Clusters on C_96_H_26_**	–31.4	–23.6	–22.1

^a^ Δ*E*_state_(Pt_6_-C_96_H_26_) = *E*_total_(triplet state) – *E*_total_(singlet state). Negative Δ*E*_state_ values indicate that the triplet state of a Pt_6_-C_96_H_26_ configuration is energetically stable relative to the singlet state.

### 2.2. Vacancy-Type Defects Formed by Removing Carbon Atoms from C_96_H_26_

**Figure 4:**
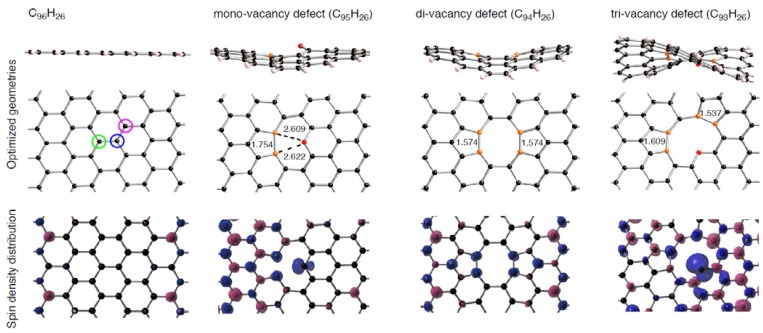
Vacancy type defects (C_96__–*n*_H_26_), constructed by removing a few carbon atoms from C_96_H_26_ where *n* ranges from 1 to 3. Parts of the optimized geometries, corresponding to the region surrounded by pink hashed lines in Figure 1, are given. Optimized bond lengths are given in Å. Their spin density distributions in the triplet state are also given. Isosurface α- and β-spins are given by pink and blue, respectively.

Prior to discussing Pt clusters adsorbed on the sp^2^ surface with vacancy-type defects, we look at how introduction of a vacancy-type defect on C_96_H_26_ changes its electronic structures. In this study, we considered the number (*n*) of carbon atoms removed from C_96_H_26_, ranging from 1 to 3. Removed carbon atoms are colored in Figure 4. First, we constructed mono-, di-, and tri-vacancy defects by removing the green atom, the green and blue atoms, and the three colored atoms, respectively. The vacancy-type defects will be denoted by C_96__–*n*_H_26_. Using the initial geometries, we obtained optimized structures for the vacancy-type defects in the triple and singlet states. Then, the energy difference between the two spin states was evaluated in each vacancy-type defect, given as Δ*E*_state_(C_96__–*n*_H_26_) in Table 2. We can see from Table 2 negative Δ*E*_state_(C_96__–*n*_H_26_) values irrespective of the number of carbon atoms removed from C_96_H_26_. The negative Δ*E*_state_(C_96__–*n*_H_26_) values indicate that each C_96__–*n*_H_26_ has energetically stable triplet state.

**Table 2:** Energy difference between the singlet and triplet states in C_96__-*n*_H_26_ (ΔE_state_ in kcal/mol) ^a^.

	***N***			
	0	1	2	3
**Δ*E*_state_**	-12.8	–14.0	–14.8	–31.4

^a^ Δ*E*_state_(C_96__–*n*_H_26_) = *E*_total_(triplet state) – *E*_total_(singlet state). Negative Δ*E*_state_ values indicate that the triplet state of a C_96__–*n*_H_26_ configuration is energetically stable relative to the singlet state.

Figure 4 also displays their optimized structures in the triplet state as well as corresponding spin density distributions. As shown in Figure 4, all optimized structures for the vacancy-type defects have some five-membered rings formed by connecting two orange atoms. There is one five-member ring in the mono-vacancy defect (C_95_H_26_), while there are two five-member rings in the other vacancy-defects (C_94_H_26_ and C_93_H_26_). Besides, removing odd-numbered carbon atoms from C_96_H_26_ generates one coordinatively unsaturated carbon atom, given by red in Figure 4. In fact, they are bound to only two neighboring atoms. The presence of vacancy-type defects perturbs significantly spin density distributions of pristine C_96_H_26_. As displayed in Figure 4, we can see radical carbon atoms around defective sites. In particular, significant spin densities were found in the tri-vacancy defect site. More interestingly, we found that the structural features of the defects have a correlation with how spin densities are distributed. On the unsaturated (red) atoms in the mono- and tri-vacancy-defects, spin densities are distributed on the carbon plane, which come from non-bonding orbitals. In contrast, spin densities on the orange atoms, which are a part of five-membered rings, are found perpendicular to the plane. The onset of radical carbon atoms at the center of the patch differentiates the defective surfaces from the pristine in terms of the interactions with Pt clusters, as will be mentioned below.

### 2.3. Platinum Clusters on Vacancy-Type Defects (C_96_H_26_) Depending on Spin States

#### 2.3.1. Singlet State

**Figure 5:**
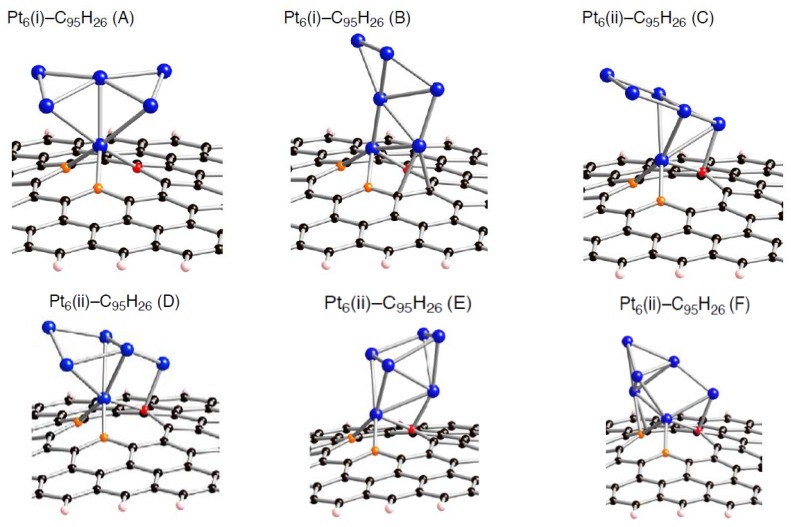
Optimized geometries for Pt_6_ cluster on the mono-vacancy-type defect in the singlet state (Pt_6_-C_95_H_26_). Optimized bond lengths are given in Table 3.

Despite the preferences of the triplet state of C_96__–*n*_H_26_ over the singlet state, let us first use the singlet state to increase our understanding of how a Pt*_k_* cluster interacts with a defective site on the sp^2^ surface. Following the previous study on interactions between a Pt_6_ cluster and pristine C_96_H_26_, we have a special interest on how the presence of a vacancy-type defect of the patch affects the interactions with a Pt_6_ cluster. In addition, we will discuss dependences of the interaction energies on size of clusters whose number of contained Pt atoms (*k*) being smaller than 6. Figure 5–Figure 7 show optimized structures for a Pt_6_ cluster adsorbed on the mono-, di-, and tri-vacancy-type defects, respectively.

Several modes for the cluster bindings were considered. For example, we obtained six optimized geometries for a Pt_6_ cluster binding into the mono-vacancy-type defect in Figure 5. The four Pt_6_-C_95_H_26_ structures displayed in Figure 5 are relatively stable in energy. In general, stable Pt*_k_*-C_95_H_26_ structures have a Pt*_k_*_-1_ moiety contained in stable Pt*_k_*_-1_-C_95_H_26_ structures. Of course, there are other possibilities for Pt*_k_* binding modes. However, our computational resource is limited, reluctantly we did not obtain other optimized geometries. We evaluated the binding energy in each configuration defined as [*E*_bind_ = *E*_total_(Pt*_k_*-C_96__–*n*_H_26_) – *E*_total_(C_96__–*n*_H_26_) – *E*_total_(Pt*_k_*)], where *k* ranges from 1 to 6 (Table 3–Table 8).

**Table 3:** Key parameters of Pt*_k_* on mono-vacancy defect (C_95_H_26_) (*k* is 1 or 6) in Figure 5. Separations of a Pt atom from orange atoms (Pt-C(orange)) and those from the red atom (Pt-C(red)). Separations of carbon atoms from a Pt atom except for the nearest Pt atom (other Pt-C), and those between the two orange atoms (C-C). Bond lengths are in Å. The *E*_bind_ and Δ*E*(Pt*_k_*) values are given in kcal/mol. Their definition was given in the text.

	*E*_bind_	Pt-C(orange)	Pt-C(red)	other Pt-C	C-C Bond	Δ*E*(Pt_k_)
**Pt_1_**	–145.7	1.953, 1.954	1.942	––	2.764	––
**Pt_6_(i)-(A)**	–152.0	1.986, 1.988	1.968	––	2.745	4.2
**Pt_6_(i)-(B)**	–146.6	1.947, 1.968	1.958	2.215	2.768	16.8
**Pt_6_(ii)-(C)**	–151.6	1.980, 1.981	1.991	2.074	2.728	17.4
**Pt_6_(ii)-(D)**	–157.1	1.983, 1.983	2.010	2.114	2.729	8.1
**Pt_6_(ii)-(E)**	–136.7	1.978, 1.976	1.991	2.200, 2.083, 2.252	2.803	15.3
**Pt_6_(ii)-(F)**	–143.4	1.986, 1.977	1.982	2.113	2.740	6.3

**Figure 6:**
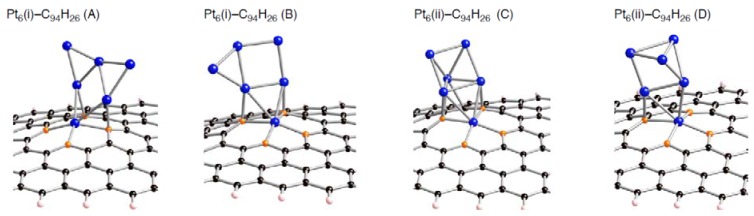
Optimized geometries for Pt_6_ cluster on the di-vacancy-type defect in the singlet state (Pt_6_-C_94_H_26_). Optimized bond lengths are given in Table 4.

**Table 4:** Key parameters of Pt*_k_* on di-vacancy defect (C_94_H_26_) (*k* is 1 or 6) in Figure 6. Separations of a Pt atom from orange atoms (Pt-C(orange)), those of carbon atoms from a Pt atom except for the nearest Pt atom (other Pt-C), and those between the two orange atoms (C-C). Bond lengths are in Å. The *E*_bind_ and Δ*E*(Pt*_k_*) values are given in kcal/mol.

	*E*_bind_	Pt-C(orange)	other Pt-C	C-C Bond	*ΔE*(Pt_k_)
**Pt_1_**	–106.1	1.999, 1.985, 1.999, 1.985	––	2.846, 2.846	––
**Pt_6_(i)-(A)**	–106.7	2.010, 2.001, 2.091, 2.117	2.088,2.033	2.810, 2.940	12.7
**Pt_6_(i)-(B)**	–106.3	2.014, 2.101, 2.109, 2.008	2.087,2.108	2.918, 2.921	14.8
**Pt_6_(ii)-(C)**	–94.5	2.014, 2.119, 2.088, 2.005	2.206,2.314, 2.084, 2.119	2.928, 2.921	17.3
**Pt_6_(ii)-(D)**	–85.1	1.994, 2.021, 2.036, 2.114	2.040	2.836, 2.941	17.9

**Figure 7:**
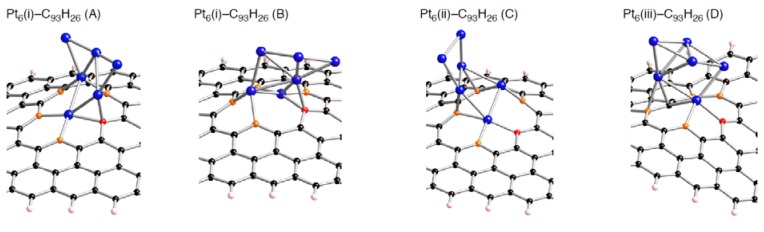
Optimized geometries for Pt_6_ cluster on the tri-vacancy-type defect in the triplet state (Pt_6_-C_93_H_26_). Optimized bond lengths are given in Table 5.

**Table 5:** Key parameters of Pt*_k_* on tri-vacancy defect (C_93_H_26_) (*k* is 1 or 6) in Figure 7. Separations of a Pt atom from orange atoms (Pt-C(orange)) and those from the red atom (Pt-C(red)). Separations of carbon atoms from a Pt atom except for the nearest Pt atom (other Pt-C), and those between the two orange atoms (C-C). Bond lengths are in Å. The *E*_bind_ and Δ*E*(Pt*_k_*) values are given in kcal/mol.

	*E*_bind_	Pt-C(orange)	Pt-C(red)	other Pt-C	C-C Bond	*ΔE* (Pt_k_)
**Pt_1_**	–162.6	2.114, 2.333, 2.097, 2.641	2.059	––	2.637, 2.641	––
**Pt_6_(i)-(A)**	–198.2	1.992, 1.959, 1.971, 1.969	2.105	2.054	2.753, 2.830	12.2
**Pt_6_(i)-(B)**	–193.4	2.006, 1.988, 1.974, 1.938	2.144	2.130	2.825, 2.746	5.9
**Pt_6_(ii)-(C)**	–188.4	1.971, 2.081, 1.970, 1.969	2.046	2.378, 2.217, 2.107	2.924, 2.922	20.0
**Pt_6_(ii)-(D)**	–142.0	2.006, 2.015	2.021	2.130, 2.231	2.710	17.9

**Figure 8:**
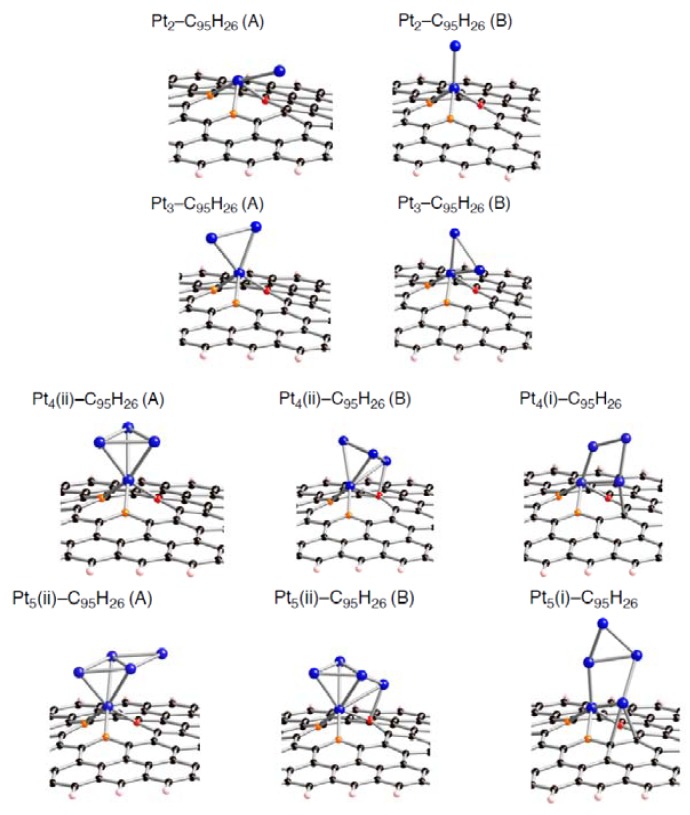
Optimized geometries for Pt*_k_* cluster (*k* = 2~5) on the mono-vacancy-type defect in the singlet state (Pt*_k_*-C_95_H_26_). Optimized bond lengths are given in Table 6.

Corresponding optimized structures for the smaller Pt*_k_* clusters adsorbed (*k* = 2, 3, 4, and 5) are also displayed in Figure 8–Figure 10.

**Table 6:** Key parameters of Pt*_k_* on mono-vacancy defect (C_95_H_26_) (*k* = 2 ~ 5) in Figure 8.

	*E*_bind_	Pt-C(orange)	Pt-C(red)	other Pt-C	C-C Bond	Δ*E*(Pt*_k_*)
**Pt_2_-(A)**	–131.7	1.964, 2.006	1.969	––	2.755	29.6
**Pt_2_-(B)**	–146.1	1.965, 1.965	1.942	––	2.743	9.0
**Pt_3_-(A)**	–132.4	1.971, 1.972	1.971	––	2.715	0.9
**Pt_3_-(B)**	–126.9	1.952, 1.976	1.962	––	2.790	2.5
**Pt_4_(ii)-(A)**	–139.7	1.983, 1.984	1.967	––	2.759	7.4
**Pt_4_(ii)-(B)**	–140.0	1.981, 1.987	1.987	2.054	2.743	7.4
**Pt_4_(i)**	–128.3	1.953, 1.966	1.960	2.227	2.757	19.8
**Pt_5_(ii)-(A)**	–140.5	1.976, 1.976	1.980	––	2.753	4.3
**Pt_5_(ii)-(B)**	–147.8	1.982, 1.982	2.002	2.105	2.741	5.4
**Pt_5_(i)**	–132.4	1.947, 1.966	1.972	2.211, 2.275	2.790	4.6

**Figure 9:**
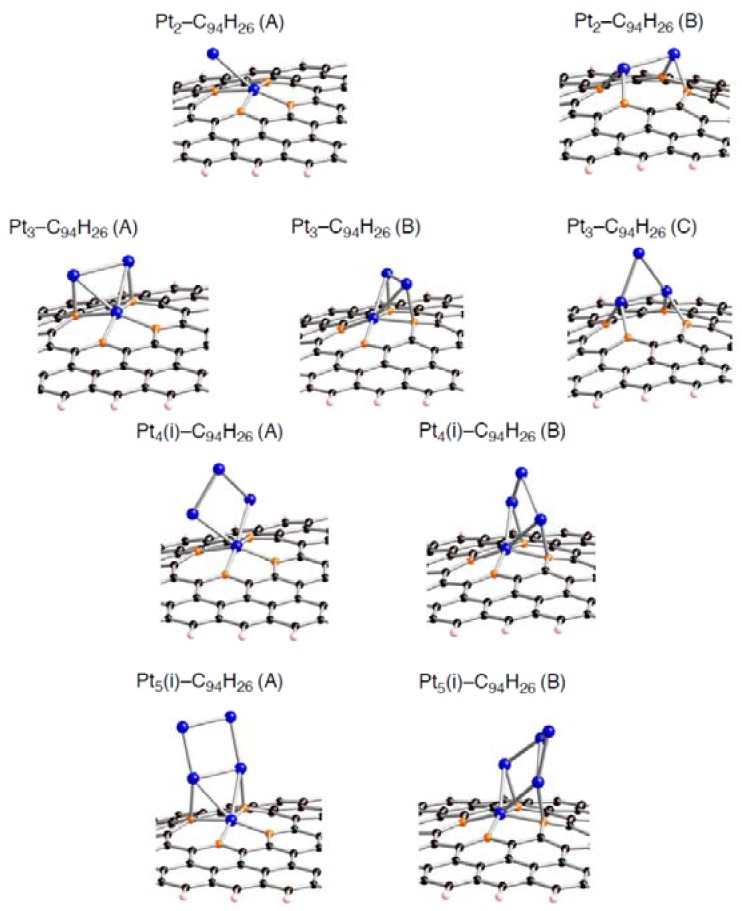
Optimized geometries for Pt*_k_* cluster (*k* = 2~5) on the di-vacancy-type defect in the singlet state (Pt*_k_*-C_94_H_26_). Optimized bond lengths are given in Table 7.

**Table 7:** Key parameters of Pt*_k_* on di-vacancy defect (C_94_H_26_) (*k* = 2 ~ 5) in Figure 9.

	*E*_bind_	Pt-C(orange)	other Pt-C	C-C Bond	Δ*E*(Pt*_k_*)
**Pt_2_-(A)**	–91.2	1.999, 2.028, 2.038, 2.066	––	2.831, 2.904	10.6
**Pt_2_-(B)**	–59.2	1.934, 1.935, 1.997, 1.997	––	2.786, 2.740	7.1
**Pt_3_-(A)**	–70.9	2.025, 2.118, 2.025, 2.119	2.045, 2.046	2.893, 2.893	9.6
**Pt_3_-(B)**	–63.2	1.990, 2.013, 2.125, 2.199	2.081, 2.082	2.791, 2.976	7.0
**Pt_3_-(C)**	–57.6	1.972, 1.987, 1.971, 1.986	––	2.690, 2.690	1.1
**Pt_4_(i)-(A)**	–84.8	2.034, 2.035, 2.101, 2.100	2.053, 2.054	2.852, 2.853	6.4
**Pt_4_(i)-(B)**	–75.8	2.004, 2.005, 2.064, 2.134	2.067, 2.066	2.786, 2.896	4.7
**Pt_5_(i)-(A)**	–93.4	2.005, 2.006, 2.085, 2.099	2.122, 2.138	2.917, 2.923	12.3
**Pt_5_(i)-(B)**	–81.3	1.991, 2.001, 2.113, 2.123	2.053, 2.082	2.806, 2.932	13.4

**Figure 10:**
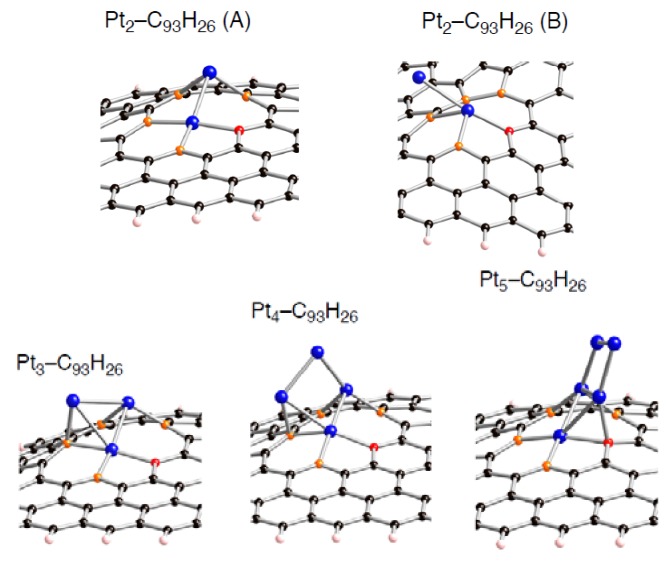
Optimized geometries for Pt*_k_* cluster (*k* = 2~5) on the tri-vacancy-type defect in the singlet state (Pt*_k_*-C_93_H_26_). Optimized bond lengths are given in Table 8.

**Table 8:** Key parameters of Pt*_k_* on tri-vacancy defect (C_93_H_26_) (*k* = 2 ~ 5) in Figure 10. Separations of a Pt atom from orange atoms (Pt-C(orange)) and those from the red atom (Pt-C(red)). Separations of carbon atoms from a Pt atom except for the nearest Pt atom (other Pt-C), and those between the two orange atoms (C-C). Bond lengths are in Å. The *E*_bind_ and Δ*E*(Pt*_k_*) values are given in kcal/mol. Their definition was given in the text.

	*E*_bind_	Pt-C(orange)	Pt-C(red)	other Pt-C	C--C Bond	Δ*E*(Pt*_k_*)
**Pt_2_-(A)**	–172.3	1.944, 1.968, 1.988, 1.950	2.038	––	2.839, 2.852	22.0
**Pt_2_-(B)**	–132.0	2.002, 1.985	2.009	2.021	2.874, 1.630	8.3
**Pt_3_**	–167.5	1.948, 1.964, 1.989, 2.124	2.021	2.067	2.999, 2.832	9.8
**Pt_4_**	–180.3	1.978, 2.068, 1.973, 1.981	2.046	2.050	2.897, 2.704	3.6
**Pt_5_**	–173.0	1.950, 1.976, 1.934, 1.997	2.099	––	2.840, 2.750	13.9

Here *E*_total_(Pt*_k_*-C_96__–*n*_H_26_) is the total energy of an optimized C_96__–*n*_H_26_ geometry, *E*_total_(C_96__–*n*_H_26_) is that of the optimized C_96__–*n*_H_26_ geometry, and *E*_total_(Pt*_k_*) is that of the optimized Pt*_k_* cluster. For the DFT calculations of the binding energies, a counterpoise correction for basis set superposition error (BSSE) was included [55]. When an *E*_bind_ value has a negative sign, the binding of a Pt cluster or the Pt atom into C_96__–*n*_H_26_ is energetically preferable. As shown in Table 3–Table 5, the calculated *E*_bind_ values in the single Pt addition are similar to those reported in [35]. These similarities verify the reliability of our DFT results.

Looking at the *E*_bind_ values, the bindings of a Pt*_k_* cluster into the sp^2^ surface are strongly facilitated by introducing vacancy-type defects. In fact, their stabilizing energies (–*E*_bind_) in stable Pt*_k_*-C_95_H_26_ structures are around 150 kcal/mol. These values are much larger than the pristine cases (about 50 kcal/mol [22]). Similar enhancement in the stabilization energies was also found in the Pt*_k_*-C_94_H_26_ and Pt*_k_*-C_93_H_26_ structures. Judging from the E_bind_ values, reactivity of vacancy-type defects toward Pt clusters declines in the order: tri-vacancy > mono-vacancy > di-vacancy. These results suggest that the tri-vacancy defect is more suitable for binding of Pt clusters into carbon surface rather than the mono- and di-vacancy defects.

From Table 3–Table 8, we see different behaviors between the three types of defect in terms of dependences of the *E*_bind_ values on Pt cluster size. Most stable Pt*_k_*-C_95_H_26_ structures except for Pt_3_-C_95_H_26_ have E_bind_ values similar to that in Pt_1_-C_95_H_26_. In contrast, the *E*_bind_ values in Pt*_k_*-C_94_H_26_ and Pt*_k_*-C_93_H_26_ are deviated from those in Pt_1_-C_94_H_26_ and Pt_1_-C_93_H_26_. When a Pt*_k_* cluster binds into the di-vacancy-defect, their E_bind_ values are smaller than the Pt_1_-C_94_H_26_ value. However, these absolute values increase gradually with an increase of the cluster size, and seem to converge to the Pt_1_-C_94_H_26_ value at *k* = 6. In the Pt*_k_*-C_93_H_26_ cases, the E_bind_ values, being around 180 kcal/mol, are always larger than the Pt_1_-C_93_H_26_ value.

To understand the energetics in the optimized Pt*_k_*-C_96__–*n*_H_26_ structures (Table 3–Table 8), let us first look at in detail geometrical features of Pt_1_-C_96__–*n*_H_26_. These key geometrical parameters in the Pt_1_-C_96__-*n*_H_26_ configurations (lengths of newly formed Pt-C bonds and of lengthening CC bonds) are listed in Table 3–Table 5.

In these tables, we can distinguish two types of the formed Pt-C bond, by whether a Pt atom binds into orange or red atoms. When the single Pt atom binds into the mono-vacancy defect, it inserts between the orange atoms in the five-membered ring, and then two P-C(orange) bonds are formed newly. As a result of the Pt addition, the separation between the orange atoms lengthens from 1.754 to 2.764 Å. At the same time, the Pt atom also coordinates to the unsaturated red atom. The binding Pt atom lifts from the sp^2^ surface, because the hole is not large enough to accommodate the Pt atom. Similar Pt lifting can be seen in the Pt_1_-C_94_H_26_ configuration, where the Pt atom inserts between orange atoms in both five-membered rings, and it breaks the connections. The degree of Pt lifting in Pt_1_-C_94_H_26_ is less significant than that in Pt_1_-C_95_H_26_, due to relatively larger hole in C_94_H_26_.

In contrast, the hole of C_93_H_26_, surrounded by ten carbon atoms, can house the Pt atom, and therefore the binding Pt atom is on the *sp*^2^ surface. Then, four Pt-C bonds are formed, accompanying the cleavage of the bonds between orange atoms in the five-membered rings. Moreover, the Pt binding into the unsaturated C atom was also seen. When a Pt*_k_* cluster binds into a vacancy-type defect, slightly longer separations of the nearest Pt atom from reactive (orange and red) atoms were found. Despite the stabilization operated between a Pt*_k_* cluster and C_96__–*n*_H_26_, slightly longer Pt-C bonds imply weakening interactions of the nearest Pt atom from the reactive carbon atoms compared with Pt_1_-C_96__–*n*_H_26_ case.

**Figure 11:**
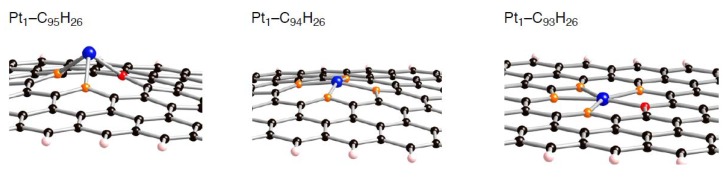
Optimized geometries for the singlet Pt atom on the mono-, di-, and tri-vacancy-type defects in the triplet state (Pt_1_-C_95_H_26_, Pt_1_-C_94_H_26,_ and Pt_1_-C_93_H_26_, respectively). Optimized bond lengths are given in Table 3–Table 5.

Compensating the weakening of the interactions, remaining Pt atoms of a clusters are additionally bound to carbon atoms of a defective site to maximize the Pt-C interactions. Then their clusters are more or less deformed from the most stable configuration in the gas-phase [56,57,58]. The degree of cluster deformation was estimated by using Δ*E*(Pt*_k_*), defined as [*E*(Pt*_k_* on surface) –* E*(Pt*_k_*)], where *E*(Pt*_k_* on surface) is the total energy of Pt*_k_* cluster taken from an optimized Pt*_k_*-C_96__–*n*_H_26_ structure and *E*(Pt*_k_*) is that of the optimized geometry for the bare Pt*_k_* cluster. Positive Δ*E*(Pt*_k_*) values in Table 3–Table 8 suggest destabilization from cluster deformation upon the interactions with a vacancy-type defect. Although we cannot find a clear correlation between E_bind_ and Δ*E*(Pt*_k_*) values, the balance between the stabilization from the Pt-C bond formation and the destabilization from the cluster deformation is a key in determining the stability. From Figure 5–Figure 7 and Figure 11, we found clear differences between Pt*_k_*-C_93_H_26_ and Pt*_k_*-C_95_H_26_ (Pt*_k_*-C_94_H_26_) in terms of the number of Pt atoms binding directly into orange atoms in the defective site to cleave connections between adjacent orange atoms.

**Figure 12:**
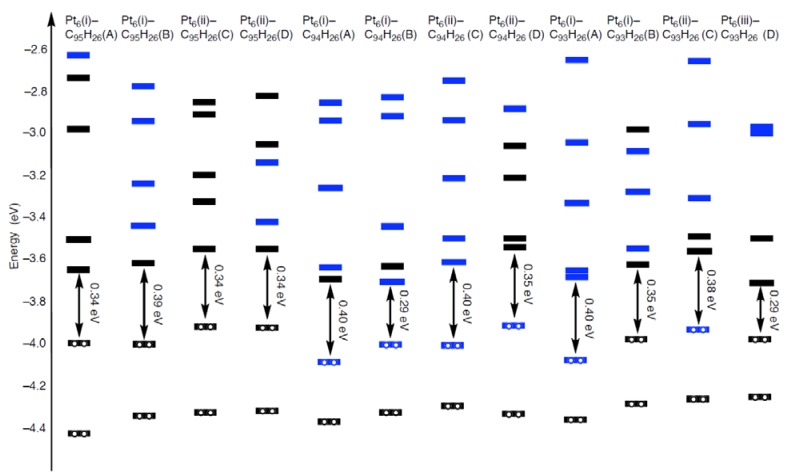
Orbital energies (eV) in the frontier orbital region of the optimized Pt_6_-C_95_H_26_, Pt_6_-C_94_H_26,_ and Pt_6_-C_93_H_26_ configurations whose structures are given in Figure 5–Figure 7. The HOMO-LUMO gaps are given. Orbitals originated from 5d(Pt) orbitals are denoted by blue bars, and those with no or less 5d(Pt) orbital contribution are denoted by black bars.

In Pt*_k_*-C_95_H_26_ (Pt_k_-C_94_H_26_), one Pt atom participates in cleaving the orange connection(s), irrespective of the cluster size. In the tri-vacancy cases containing larger ten-membered ring, two Pt atoms in a cluster bind to the defective site to split two orange connections. The accommodation of two Pt atoms cannot be seen in the Pt_1_-C_94_H_26_ structure, and thus the significant enhanced stabilization in Pt*_k_*-C_93_H_26_ is understandable. Moreover the acceptability of the ten-membered-ring to trap Pt atoms differentiates C_93_H_26_ from C_95_H_26_ and C_94_H_26_ in terms of their reactivity toward Pt*_k_* clusters. Due to the strong interactions between a Pt_6_ cluster and a vacancy-type defect, we can see unique orbital features, which cannot be seen in C_96__–*n*_H_26_ (Figure 12).

**Figure 13:**
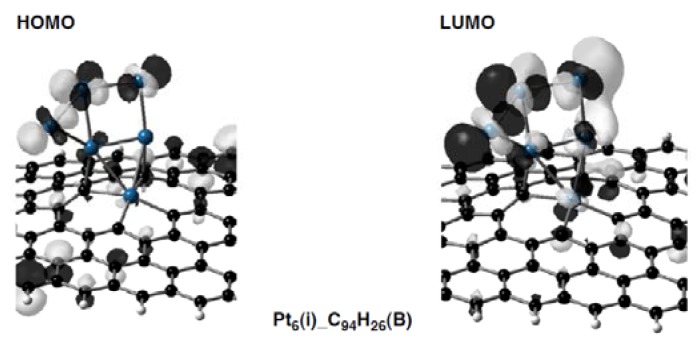
Frontier orbitals (the HOMO and LUMO) in the Pt_6_(i)-C_94_H_26_(B) configuration (Figure 6) are given as a representative Pt_6_-C_96__–*n*_H_26_ configuration.

In fact, 5d(Pt)-based orbitals, given by blue bars in Figure 12, appear in the frontier orbital regions of the Pt_6_-C_94_H_26_ and Pt_6_-C_93_H_26_ configurations. As the most striking case, we can see in Figure 13 that the Pt_6_(i)-C_94_H_26_(B) configuration has the HOMO and LUMO consisting of 5d(Pt) orbitals. On the other hand, levels of 5d(Pt)-based orbitals in the Pt_6_-C_95_H_26_ strongly depend on their cluster-shape. In the Pt_6_(i)-C_95_H_26_(A) and Pt_6_(ii)-C_95_H_26_(C) configurations, such 5d(Pt)-based orbital lies larger than 1.3 eV above the LUMO, whereas the LUMO+1 consists of 5d(Pt)-based orbitals in the other Pt_6_-C_95_H_26_ configurations.

#### 2.3.2. Triplet State

As shown in Figure 12, the all optimized geometries in the single state have relatively small HOMO-LUMO gaps (0.29 ~ 0.40 eV). Thus, higher spin states can be energetically stable relative to the singlet states. Along the assumption, we obtained their triplet states, and estimated the energy difference between the two spin states (ΔE_state_(Pt_6_-C_96__–*n*_H_26_)), as tabulated in Table 9.

**Table 9:** Energy difference between the singlet and triplet states in the Pt_6_-C_96__–*n*_H_26_ configurations (ΔE_state_ in kcal/mol) ^a^.

	Pt_6_(i)-(A)	Pt_6_(i)-(B)	Pt_6_(ii)	Pt_6_(iii)
**Clusters on C_95_H_26_**	–14.3	–17.8	–14.0	^b^
**Clusters on C_94_H_26_**	–23.3	–16.2	–23.3	–38.5
**Clusters on C_93_H_26_**	–15.0	–23.7	–13.7	^b^

^a^ Δ*E*_state_(Pt_6_-C_96__–*n*_H_26_) = *E*_total_(triplet state) – *E*_total_(singlet state). Negative Δ*E*_state_ values indicate that the triplet state of a Pt_6_-C_96__–*n*_H_26_ configuration is energetically stable relative to the singlet state. ΔE_state_ in kcal/mol; ^b^ we could not obtain the optimized geometry in the triplet state.

**Figure 14:**
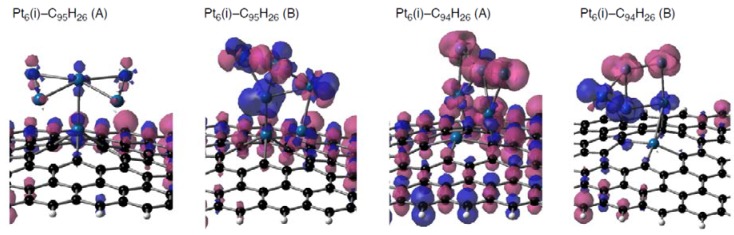
Spin density distributions of representative Pt_6_-C_96__–*n*_H_26_ configurations (Pt_6_(i) cluster on C_95_H_26_ or C_94_H_26_ in two binding fashions, displayed in Figure 5 and Figure 6). Isosurface α- and β-spins are given by pink and blue, respectively.

**Figure 15:**
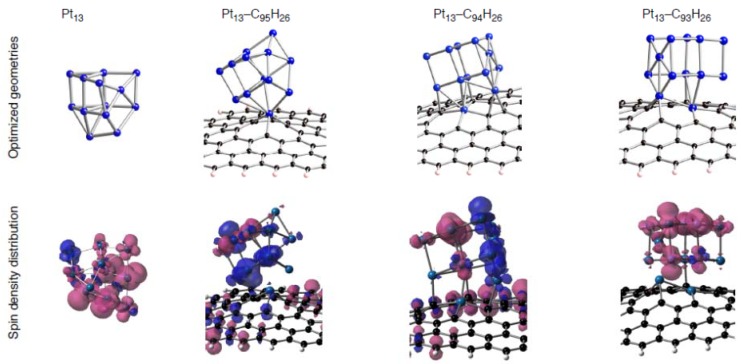
Spin density distributions of representative Pt_13_-C_96__–*n*_H_26_ configurations. Isosurface α- and β-spins are given by pink and blue, respectively.

Table 9 shows that their triplet spin states are energetically stable relative to the singlet states, as expected. According to DFT calculations, most Pt_6_-C_96__–*n*_H_26_ configurations have radical Pt_6_ clusters on defective graphene patches As representative cases, spin density distributions on the Pt_6_(i) cluster binding into C_95_H_26_ or C_94_H_26_ are shown in Figure 14. Figure 14 shows that substantial spin densities appear on the adsorbed clusters in the three Pt_6_-C_96__–*n*_H_26_ configurations (Pt_6_(i)-C_95_H_26_(B), and two Pt_6_(i)-C_94_H_26_ configurations), whereas the Pt_6_(i)-C_95_H_26_(A) configuration does not have such radical cluster due to the absence of 5d(Pt)-based frontier orbitals (Figure 12). In the three configurations with radical clusters, a variety of the spin density distributions was found. In the Pt_6_(i)-C_95_H_26_(B) and Pt_6_(i)-C_94_H_26_(B), the spin density distributions are strongly deviated from those in the bare Pt_6_ cluster, whereas the Pt_6_(i)-C_94_H_26_(B) has similar distributions. Furthermore, we found a relationship between spin densities of Pt clusters and those on the defective sp^2^ surface. When spin density distributions of the Pt cluster of a Pt_6_-C_96__-*n*_H_26_ configuration are (not) similar to the bare case, spin densities are (not) delocalized over the carbon surface. Similar tendencies were found in spin density distributions of the stable triplet Pt_13_-C_96–*n*_H_26_ configurations [59], because radical Pt clusters exist on the defective graphene patches as shown in Figure 15.

Finally, let us compare the spin density distributions on Pt_6_ clusters on defective sp^2^ surfaces (Figure 14) with the pristine case (Figure 2). As mentioned above, spin density maps on Pt_6_ cluster on pristine C_96_H_26_ are similar to those in the bare cluster. However, such similarity cannot be always found in the defective graphene cases. The different tendencies come from whether underlying carbon atoms have radical characters or not in Figure 2 and Figure 4. In the pristine patch, underlying carbon atoms do not have radical characters, and they cannot perturb spin density distributions of the absorbed cluster. In contrast, unpaired electrons exist on underlying carbon atoms in the defective sites, and thus spin density distributions of the adsorbed clusters are modulated by the Pt-C interactions. Therefore, perturbation of the radical sp^2^ surface by introduction of vacancy-type defects can change characters of adsorbed Pt clusters.

The DFT findings are important in the catalytic activity of Pt clusters on sp^2^ surface, because the supported clusters can serve as active site for catalytic reactions. For example, if radical Pt clusters exist on carbon surface, it can cleave the H-H bond of hydrogen molecules in a homolytic manner. Otherwise, the H-H bond is activated by the clusters via a non-radical mechanism in [22]. Thus, the DFT calculations propose that one can change chemical reactivity of Pt clusters on graphene patches by introducing of vacancy-type defects on the surface.

## 3. Experimental

To investigate interactions of Pt clusters with C_96_H_26_, we carried out DFT calculations implemented in the Gaussian 03 and 09 program packages [60,61]. Adsorbed clusters that we considered consist of one, six, and thirteen Pt atoms. To perform the calculations, a hybrid Hartree–Fock/density functional theory method, B3LYP [62,63,64,65,66] was used. The B3LYP method consists of the Slater exchange, the Hartree–Fock exchange, the exchange functional of Becke [62,63,64], the correlation functional of Lee, Yang, and Parr (LYP) [65], and the correlation functional of Vosko, Wilk, and Nusair (VWN) [66]. In general the hybrid B3LYP method has been reported to provide excellent descriptions of various properties. The Gaussian-type basis set we used for the C and H atoms is 6-31G* [67], and that for the Pt atom is the quasi-relativistic effective core potential RECP and valence basis sets recommended by Stuttgart group (SDD) [68]. The SDD RECP is adjusted to total valence energies of a multitude of atomic references states, which are quantum mechanical observables [68]. As indicated in the previous papers [69,70,71,72,73,74,75,76], the B3LYP/6-31G* calculations correctly reproduce experimental data for C_60_, especially its IR and Raman vibrational frequencies. According to the theoretical report by Nova *et al*. [77], the method of our choice (B3LYP/SDD + 6-31G*) is appropriate to reproduce experimental values in terms of Pt-C bonds in Pt complexes [77]. The computational method is also suitable to study transition metals adsorbed on graphene. In fact, Pt-C bond lengths obtained from the B3LYP/SDD + 6-31G* calculations fall in the range reported from other theoretical reports [24,30].

## 4. Conclusions

Density functional theory (DFT) B3LYP calculations were employed to investigate the adsorption of Pt*_k_* cluster (*k* is 1–6, and 13) into a nanometer-size graphene patch (C_96_H_26_) with or without vacancy-type defects. According to the DFT calculations, removing a few carbon atoms (*n*) from C_96_H_26_ results in the formation of five-membered rings as well as coordinatively unsaturated carbon atoms. Introduction of a vacancy-type defect on C_96_H_26_ strongly affects spin density distributions in its stable triplet state. Although spin densities appear only on edge carbon atoms of the triplet C_96_H_26_ structure, defective graphene patches have radical carbon atoms at the center where the reactive carbon atoms exist. These spin density distributions differentiate characters of Pt clusters adsorbed on defective graphene patches from those on the pristine. According to the DFT calculations, spin density maps of Pt clusters on C_96_H_26_ are similar to those of the corresponding bare clusters. In contrast, Pt clusters interact strongly with radical carbon atoms in defective graphene patches, and thus spin density distributions of the adsorbed Pt clusters are usually deviated from the bare cases. Consequently, DFT calculations propose that characters of Pt clusters adsorbed on the sp^2^ carbon surface can be modulated by introducing vacancy-type defects.
